# Integrated analysis of transcriptomic datasets to identify placental biomarkers of spontaneous preterm birth

**DOI:** 10.1016/j.placenta.2022.03.122

**Published:** 2022-04-04

**Authors:** Nasim C. Sobhani, Rachel Mernoff, Mosana Abraha, Chinomnso N. Okorie, Leticia Marquez-Magana, Stephanie L. Gaw, Joshua F. Robinson

**Affiliations:** a Center for Reproductive Sciences and Department of Obstetrics, Gynecology & Reproductive Sciences, University of California San Francisco, 499 Illinois Street, San Francisco, CA, 94158, USA; b Division of Maternal Fetal Medicine, Department of Obstetrics, Gynecology & Reproductive Sciences, University of California San Francisco, 1825 Fourth Street, San Francisco, CA, 94158, USA; c UC Berkeley-UCSF Joint Medical Program, University of California, Berkeley School of Public Health and University of California San Francisco School of Medicine, 533 Parnassus Avenue, San Francisco, CA, 94143, USA; d Integrated Biomedical Engineering and Health Sciences, McMaster University, 1280 Main Street W, Hamilton, ON L8S 4L8, Canada; e Department of Biology, San Francisco State University, 1600 Holloway Ave, San Francisco, CA, 94132, USA

**Keywords:** Placenta, Transcriptome, Preterm birth, Pregnancy, Epigenetics, Stress

## Abstract

**Introduction:**

Preterm birth (PTB) remains the leading cause of neonatal morbidity and mortality in the United States. The mechanisms underlying spontaneous PTB (SPTB) involve multiple physiological processes and molecular transformations at the level of the placenta. This study aimed to identify consistent molecular correlates in the placenta linked with SPTB by cross-examining publicly available transcriptomic datasets within two publicly available repositories.

**Methods:**

The National Center for Biotechnology Information and the European Bioinformatics Institute were queried, and relevant datasets were independently normalized, and then merged based on similarity in design. Differentially expressed genes between SPTB and term delivery (TD) were identified using a fixed effects linear model (p < 0.0001) and were evaluated for enrichment of biological processes and pathways. In general, global signatures associated with SPTB were unique to each study.

**Results:**

A total of three datasets were used in the meta-analysis to assess the placental transcriptome in SPTB (11 samples) as compared to TD (15 samples). We identified 174 differentially expressed genes consistently correlated with SPTB across all studies, including previously proposed and new candidate biomarkers of SPTB. Differentially expressed genes were significantly enriched for master regulatory pathways relevant to placental development and disease, including chromatin organization and cellular response to stress.

**Discussion:**

Identification of differentially expressed genes and associated pathways across multiple studies may identify transcriptomic biomarkers that can be applied in clinical investigations of SPTB and provide researchers enhanced insight into the underlying etiologies of SPTB.

## 1. Introduction

Affecting approximately 10% of pregnancies in the United States, preterm birth (PTB) < 37 weeks gestation is the leading cause of neonatal morbidity and mortality in this country and contributes to significant medical, psychosocial, and economic burden for affected patients and families [1,2]. Approximately two-thirds of the cases of PTB are spontaneous in nature (SPTB), defined as delivery preceded by the spontaneous onset of labor. Many clinical risk factors for SPTB have been identified, including a prior history of SPTB, cervical shortening in the second trimester, tobacco use, multifetal gestation, and intrauterine infection [3,4]. Importantly, however, the vast majority of SPTB occur in the absence of identifiable risk factors.

In light of the poor performance of clinical risk factors in predicting SPTB, efforts have been made to identify potential biomarkers of SPTB. As the maternal-fetal interface, the placenta has appropriately been the focus of many SPTB investigations [5]. Investigators have proposed a host of biophysical, biochemical, immunologic, microbiologic, molecular, metabolomic, and exosomal biomarkers [6]. However, the findings from individual studies are infrequently replicated, and a consistent, predictive, placental signature for SPTB has yet to be identified. As a result, these biomarkers have not been incorporated into clinical practice.

Integration of previously published and publicly available omic datasets is a promising approach to identify the most robust signals associated with specific disease states. This method allows one to enhance sample size and to focus on the most significant and consistent changes in biomarkers, by amplifying disease-specific signals and diminishing confounding signals. This study was designed to examine the transcriptomic signature of the placenta in SPTB compared to term delivery (TD), using independent and aggregate analyses of publicly available datasets.

## 2. Materials and methods

### 2.1. Identification of transcriptomic datasets

We acquired transcriptomic datasets from two public repositories, the National Center for Biotechnology Information (NCBI) and the European Bioinformatics Institute (EBI). We identified studies relevant to PTB and human placenta by searching with following terms: “human” AND “placenta” AND “preterm” (final search on January 24, 2020). We performed a secondary *ad hoc* review of datasets to assure studies met the first-pass criteria: 1) Affymetrix or Illumina array or RNA-seq platforms; and 2) included a minimum of two biological replicates per group. We summarized datasets based on platform, population size, tissue analyzed, and control group (Supplemental Table 1). SPTB was defined as delivery <37 weeks preceded by the spontaneous onset of preterm labor, and TD was defined as delivery ≥37 weeks gestation preceded by either spontaneous or induced labor. We excluded samples from medically indicated PTB, from unlabored cesarean deliveries, and from pregnancies affected by other obstetric complications (e.g., preeclampsia and/or fetal growth restriction) if described.

### 2.2. Processing of datasets and identification of differentially expressed genes

We identified three datasets examining placental transcriptomic differences in SPTB vs. TD. Microarray datasets were independently processed using BRB Arraytools 3.6 [7]. Raw values were normalized via the Robust Multi-array Average (RMA) algorithm and annotated using the Affymetrix database. We applied two approaches to identify differentially expressed (DE) genes in placentas of SPTB cases versus TD controls. First, a one-factor generalized linear model (GLM) was independently applied to each dataset to determine significance of DE genes between SPTB and TD and to determine the overlap in commonly dysregulated genes (uncorrected, p < 0.05). Second, normalized data were integrated using the Official Gene Symbol (OGS), and a two-factor GLM was applied to assess the effects of SPTB while controlling for study, with a cutoff of p < 0.0001 (uncorrected; false discovery rate <1%). In the case of multiple probes per gene, the one with the lowest p-value (i.e., the one with the most significant changes between SPTB and TD within each independent study) was used for comparison purposes. Within each study, we calculated average log2 fold differences in expression between SPTB vs. TD placentas. Hierarchical clustering of fold change values for DE genes was determined using average linkage and Euclidean distance [8]. To examine the variance and fold change in a subset of DE genes (top 20 most significant; p ≤ 8.3E-6), irrespective of study, we adjusted intensity values to the average TD within each study and plotted values via PRISM.

### 2.3. Enrichment of GO biological processes in DE genes

We utilized the Database for Annotation, Visualization, and Integrated Discovery (DAVID, version 6.8, Laboratory of Human Retro-virology and Immunoinformatics, Frederick, MD) to identify overrepresented biological processes in genes discovered to be DE between SPTB and TD placentas [9]. GO analysis was performed on all levels, but only categories at “level 4” were reported, in order to reduce redundancy of terms of similar function. Within the GO hierarchy, “level 4” represents categories of moderate to high specificity. We defined significant enrichment as p < 0.01 and a minimum of 5 DE genes changes within each biological process. Terms were grouped based on themes following the GO hierarchical system [10]. Due to the relevancy of chromatin regulation and cellular stress in pregnancy complications, we explored differences in the expression of genes associated with enriched terms “chromosome organization” and “cellular response to decreased oxygen levels” between SPTB and TD via hierarchical clustering (average linkage and Euclidean distance [8]).

### 2.4. Transcription factor binding site enrichment analysis of DE genes

We evaluated for overrepresentation of DNA sequences in promoter regions of DE genes corresponding with known transcription factor binding sites (TFBS) using OPOSSUM [11]. Sequence information was available for comparison of 96% of DE genes (167/174 genes). We identified enriched motifs as (1) 2000 bases upstream of the transcription start site (TSS) of each gene; (2) an identification score of ≥0.4 and matrix score threshold of ≥0.85 (default parameters); (3) a Fisher score of p ≥ 20, a statistical indicator of the enrichment of the genes/sequences that contain the TFBS in the DE gene subset compared to the total genome; and (4) a Z-score of ≥ 10, a statistical indicator of the enrichment of the TFBS in the DE subset as compared to the total genome.

## 3. Results

### 3.1. Identification and summary of transcriptomic datasets

On initial inquiry, 28 NCBI studies and 15 EBI studies were identified. After review of these 43 studies, only eight NCBI studies and four EBI studies met the first pass inclusion criteria. Of these, four utilized a non-term control group (e.g., preterm preeclampsia, preterm premature rupture of membranes, infected PTB) and five analyzed tissue other than whole placenta (Supplemental Table 1). The remaining three studies were included in the subsequent transcriptomic analysis. The combined total sample from these three studies included n = 11 SPTB and n = 15 TD controls (Table 1). Study A (GEO accession: GSE98224) samples were provided from Mount Sinai Hospital in Ontario, Canada through the Research Centre for Women’s and Infants’ Health (RCWIH) [12]; Study B (GSE18809) from Prince of Wales Hospital, Hong Kong [13]; and Study C (GSE73685) from patients at the University of Alabama at Birmingham, University of Texas Medical Branch at Galveston, and University of Utah [14]. Studies A and C reported ascertainment of gestational age using sonographic estimation with or without menstrual dating. Study B did not specify method of ascertainment of gestational age, but the authors noted that they purposely defined preterm <34 weeks and term ≥39 weeks to prevent misclassification.

### 3.2. Identification of DE genes between SPTB and TD

**Fig. 1.**
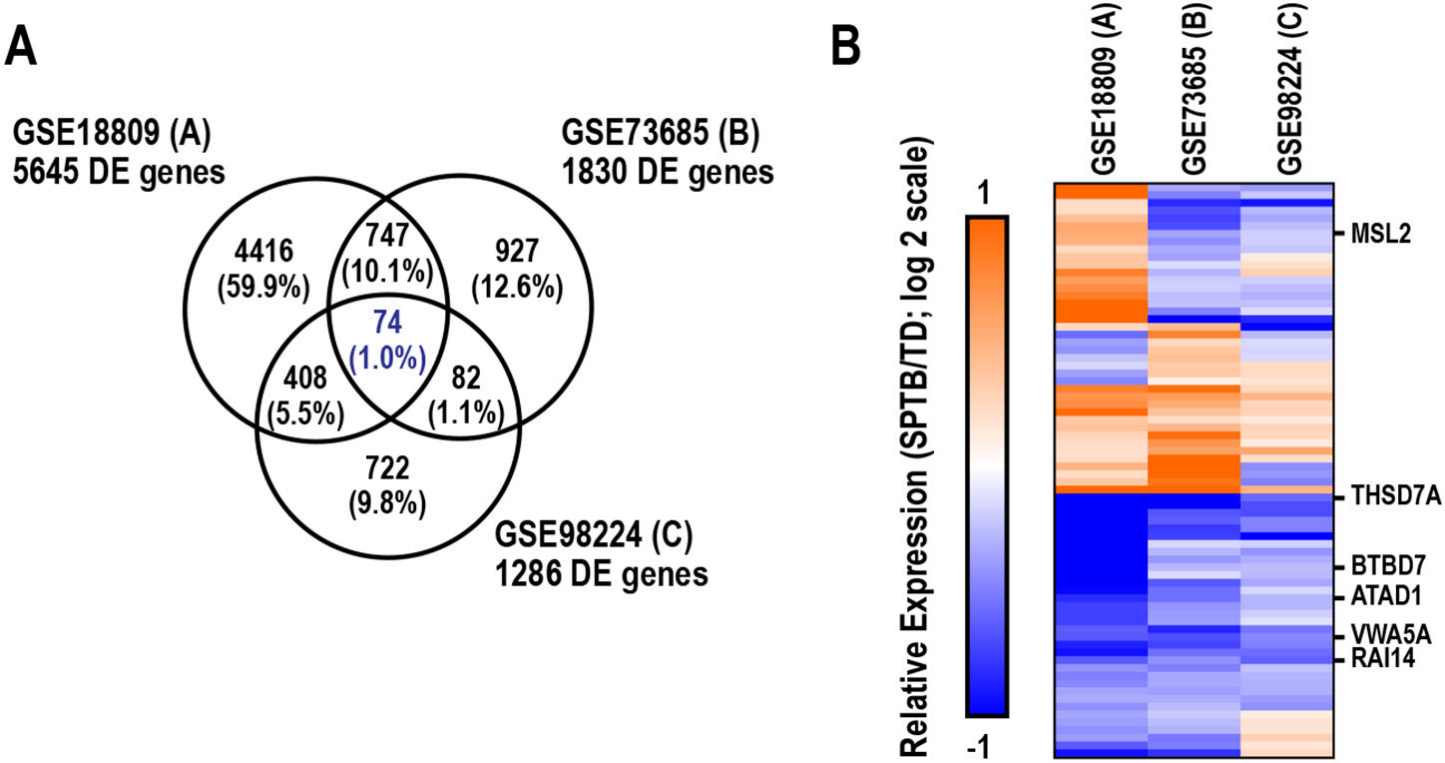
Independent analysis and overlap of differentially expressed (DE) genes in human placental transcriptomic studies examining differences between spontaneous preterm birth (SPTB) and term delivery (TD). (A) Distribution of DE genes independently identified in each study based on p < 0.05, with Venn diagram demonstrating overlap in DE genes across the three studies. (B) Heatmap of common DE genes. Labeled genes are those that were commonly DE with p < 0.01 in all three studies.

We applied two statistical approaches: 1) identification of DE genes within each study and examination of the overlap in DE genes across the three studies; and 2) integration of the three datasets and identification of DE genes using a two-factor GLM. When independently evaluated, thousands of genes (i.e., 1286 to 5645 genes) were identified as DE in placentas of SPTB as compared to TD in each study when using criteria with limited constraints (uncorrected p < 0.05; Fig. 1A). However, only 74 genes were identified to be commonly DE in all three studies. Of this subset, only 53% were commonly dysregulated (up or down regulated across all three studies) (Fig. 1B). More conservative criteria yielded even fewer DE genes in common (e.g., only 6 genes commonly DE with p < 0.01, denoted in heatmap in Fig. 1B). In general, these results suggest that transcriptomic signatures of the placenta between SPTB and TD are highly-study dependent.

**Fig. 2.**
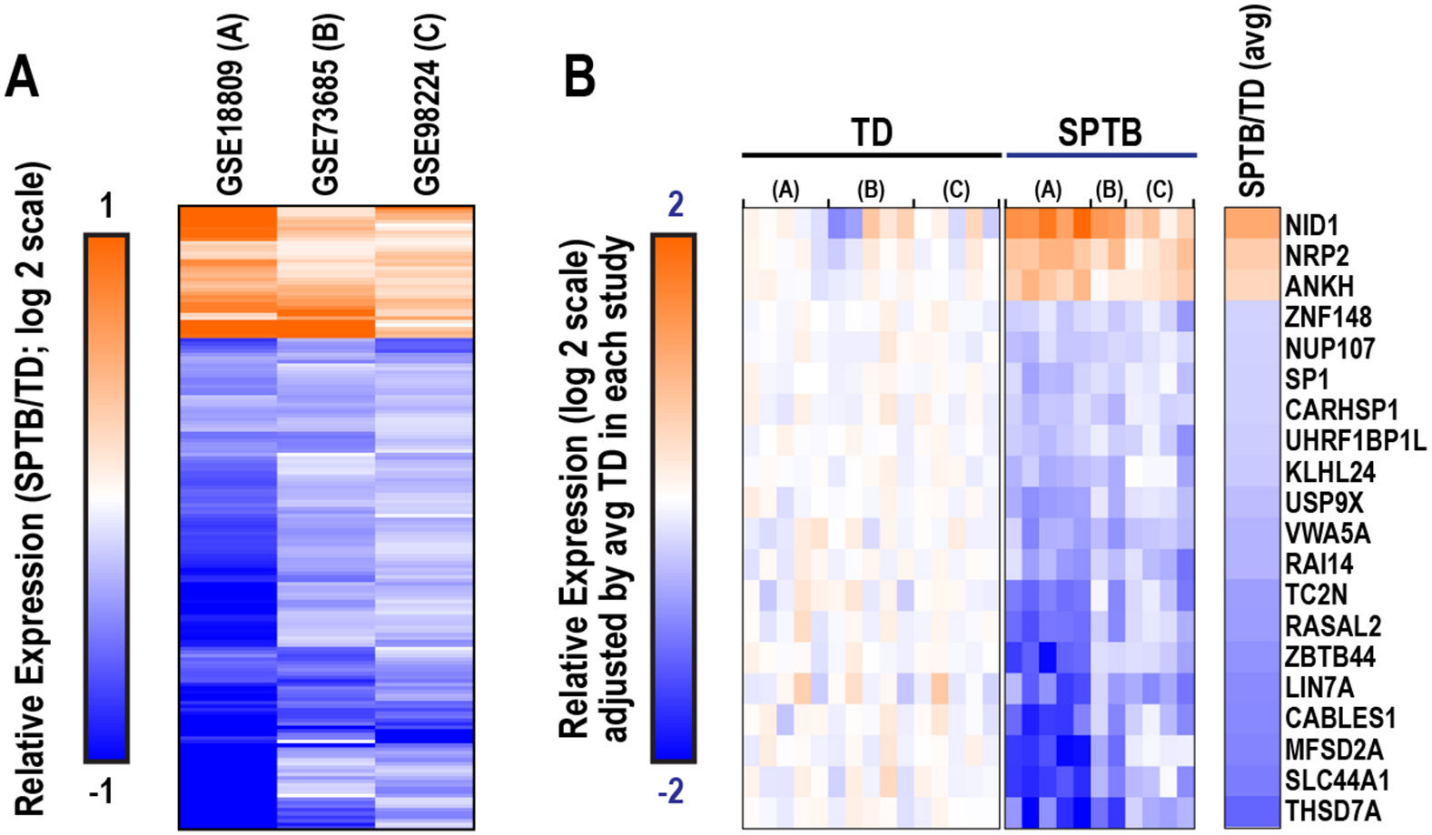
Aggregate analysis and identification of differentially expressed (DE) genes in human placental transcriptomic studies examining differences between spontaneous preterm birth (SPTB) and term delivery (TD). (A) Hierarchical clustering plot of 174 genes identified to be DE (p < 0.0001). (B) Relative expression of the top 20 most statistically significant DE genes (p ≤ 8.3E-6) in SPTB and TD placentas. Expression values were adjusted by the average expression of the TD group within each dataset to enable relative comparisons across studies. Average fold change in expression indicates relative differences in expression between SPTB and TD across all three studies.

With the second approach of integrating the datasets prior to analyzing for differential expression, we identified 174 DE genes using more conservative criteria (uncorrected, p < 0.0001; FDR<1%). Of these, 37 were upregulated and 137 were downregulated in SPTB (Fig. 2A). The overwhelming majority of DE genes (173 of 174 genes; 99.4%) trended in a similar direction across the three individual studies in relation to SPTB versus TD. Based on p-value, the top 20 most DE genes were *NID1, NRP2, ANKH, ZNF148, NUP107, SP1, CARHSP1,* UHRF1BP1L, *KLHL24, USP9X, VWA5A, RAI14, TC2N, RASAL2, ZBTB44, LIN7A, CABLES1, MFSD2A, SLC44A1,* and *THSD7A* (p ≤ 8.3E-6; Fig. 2B).

### 3.3. Enrichment of biological processes in DE genes

**Fig. 3.**
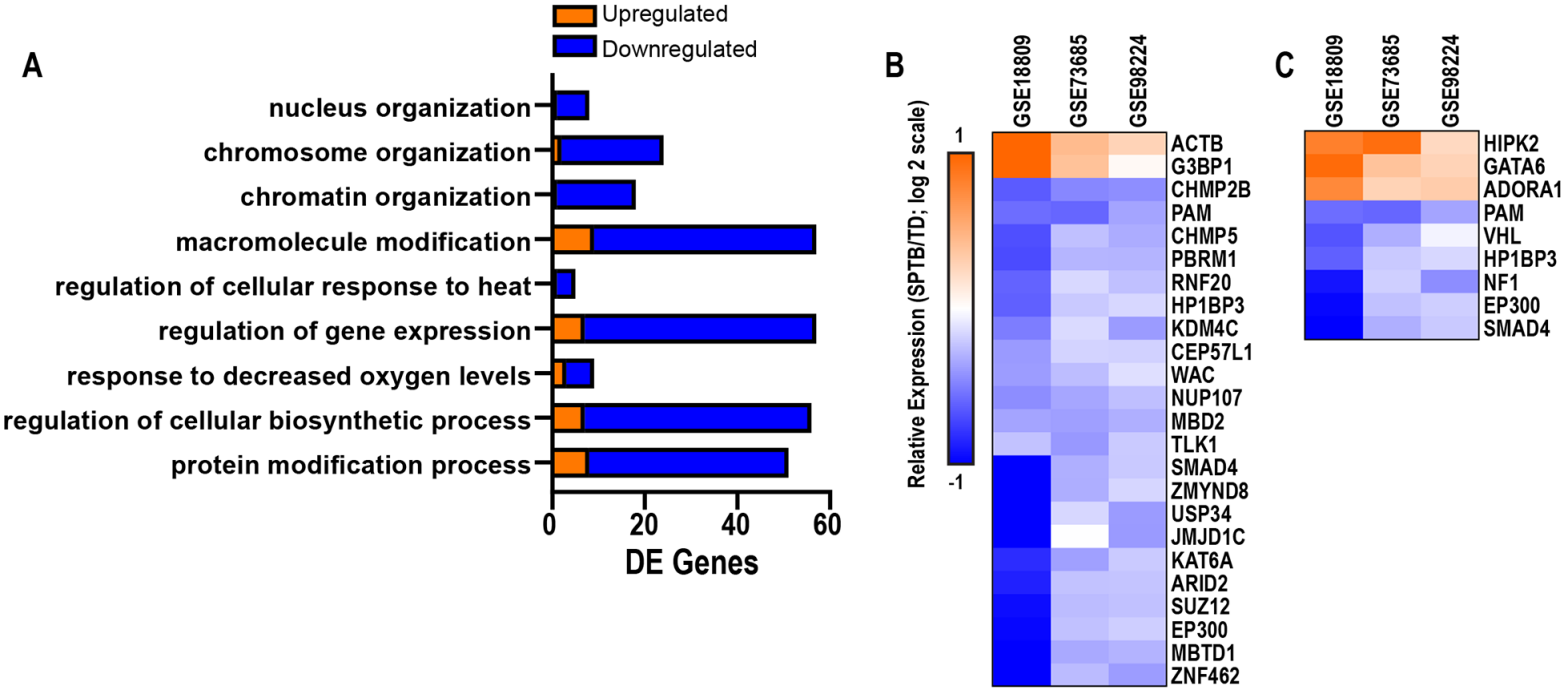
Enrichment analysis of biological processes in differentially expressed (DE) genes in spontaneous preterm birth (SPTB) versus term delivery (TD). (A) Overrepresented biological processes (<0.01 and a minimum of 5 DE genes). Hierarchical clustering plots of DE genes associated with (B) chromosome organization and (C) cellular response to decreased oxygen levels.

DE genes identified were significantly enriched for biological processes related to chromosome organization (i.e., chromosome organization, chromatin organization, nucleus organization), macromolecule modification, regulation of gene expression, regulation of cellular biosynthetic process, and cellular stress pathways (i.e., regulation of cellular response to heat, response to decreased oxygen levels; Fig. 3A). A total of 24 DE genes were associated with chromosome organization (p = 0.001), and the majority of these (22 of 24) were downregulated with SPTB (Fig. 3B). Upregulated genes included *ACTB* and *G3BP1*, while downregulated genes included *SUZ12, RNF20, ZNF462, PBRM1, SMAD4, NUP107, KDM4C, ZMYND8, MBD2, USP34, HP1BP3, JMJD1C, MBTD1, CEP57L1, KAT6A, CHMP2B, WAC, EP300, TLK1, ARID2, PAM,* and *CHMP5*. A total of 9 DE genes were associated with cellular response to decreased oxygen levels (p = 0.008), including 3 upregulated in SPTB (*HIPK2, GATA5,* and *ADORA1*) and 6 downregulated in SPTB (*PAM, VHL, HP1BP3, NF1, EP300,* and *SMAD4*) (Fig. 3C). These analyses highlight specific transcriptomic pathways dysregulated in SPTB as compared to TD.

### 3.4. Transcription factor binding site enrichment analysis of SPTB and DE genes

We interrogated upstream promoter sequences of DE genes between SPTB and TD to identify enrichment of known motifs corresponding with master regulators (transcription factors (TFs)) that may influence differences in transcriptional signatures between the two groups (Table 2). Within the DE gene subset, we identified 13 motifs corresponding with specific TFs that were significantly enriched, including homeobox (e.g., *NKX2-5, PRRX2, HOXA5*), forkhead (e.g., *FOXA2, FOXA1, FOXQ1*), High Mobility Group (e.g., *SOX17*), TATA-binding (*TBP*), Arid (*ARID3*), Rel (*NFATC2*), and GATA (*GATA1*) TF family members. The most overrepresented TF identified was *NKX2-5*; 120 of 174 DE genes were discovered to contain motifs specific to *NKX2-5* (Z = 20.9; Fisher = 28.7). Using Protein Atlas [15], we determined cell types predicted to express the proposed TFs as well as the placental cell type with the highest expression. These analyses indicated that the majority of TFs identified here are expressed highest in the mesenchymal (fibroblasts) or vascular (endothelial) cells, with the exception of *ARID3A* (trophoblasts) and *NFATC2* (Hofbauer cells), suggesting particular placental cell types involved in the SPTB transcriptional phenotype.

## 4. Discussion

### 4.1. Principal findings

Independent analysis of publicly available transcriptomic datasets comparing SPTB to TD demonstrated limited overlap of 74 common DE genes, representing only ~1% of all DE genes identified in the individual studies. By aggregating data and utilizing a statistical model to control for study-dependent effects, we were able to identify 174 DE genes across the three studies. These were consistently up- or down-regulated across the three studies evaluated and were significantly enriched for master regulatory pathways including chromatin organization and cellular response to stress. Interrogation of promoter genomic sequences of DE genes implicated specific TF as potential master regulators in SPTB such as *NKX2-5*. In addition to validating previously proposed biomarkers of SPTB, our analysis also identified new candidate biomarkers for SPTB.

### 4.2. Datasets utilized in meta-analysis

Several studies have examined the placental transcriptome using a variety of unique experimental designs and approaches to determine underlying mechanisms and biomarkers of SPTB. While each of these studies has provided valuable information in terms of dysregulated genes and processes in SPTB, generalizability of the results is lacking due to the complexity in SPTB mechanisms and diversity in study design and analysis. In this study, we narrowed our efforts to define genes DE between SPTB and TD across similar studies. We excluded samples from medically indicated PTB, unlabored cesarean deliveries, and pregnancies affected by other obstetric complications (e.g., preeclampsia and/or fetal growth restriction) if described. In total, we utilized a subset of samples from three datasets that met our predefined inclusion criteria. Leavey and colleagues performed Affymetrix microarray on 157 placentas from a Canadian biospecimen bank, including 25 from normotensive PTB and 28 from normotensive TD (of which n = 4 and n = 5, respectively, had data available in a public repository). The authors focused on identification of different phenotypes of preeclampsia and thus did not comment specifically on genes that were DE between the normotensive PTB cases and the normotensive TD controls [11]. Chim and colleagues evaluated placental gene expression profiles in SPTB and TD in placentas (n = 20) collected after vaginal delivery [12]. In total, 426 DE genes (*p* < 0.05) were identified, including genes involved in acute inflammatory response, RNA stabilization, and extracellular matrix binding. Bukowski and colleagues analyzed the transcriptomes of multiple gestational matrices (i.e., myometrium, decidua, amnion, chorion, placenta, fetal blood, and maternal blood) of four phenotypes: SPTB (n = 8), medically indicated PTB (n = 10), TD preceded by spontaneous labor (n = 7), and TD without labor (n = 10) [13]. The authors described 50 distinct genes and non-coding RNA sequences unique to SPTB samples, although the accuracy of classification of SPTB samples based on this transcriptomic signature was only 47%. These 50 DE genes represent pathways of infection, inflammation, and immune rejection. From these three studies, we acquired placental transcriptomic datasets exclusively of SPTB (n = 11) and TD with labor (n = 15).

### 4.3. Study-dependent transcriptomic signatures

Initially, we followed a standard gene expression analysis pipeline by processing each dataset and evaluating for DE genes in each of the studies independently. In general, a lack of concordance was observed across the three studies; only 74 genes overlapped, despite identification of >1200 genes in each of the three studies. Furthermore, common DE genes in this context did not necessarily display common trends in dysregulation between SPTB and TD; only 53% of DE genes were commonly dysregulated. This limited overlap between transcriptomic studies has previously been described. For example, among 6444 genes identified in 53 studies examining placental tissue in term and preterm deliveries, only 2 genes were consistently found in more than 10 studies [16]. In a similar meta-analysis examining the placental transcriptome of preeclampsia, less than 8% of the 6122 DE genes identified in the individual studies were also detected as DE genes in the meta-analysis [17]. Importantly, these authors also identified 147 additional DE genes on meta-analysis that were not identified in any of the primary studies. These findings highlight the variability and non-reproducibility of individual gene expression studies, while also emphasizing the utility and efficiency of an integrated meta-analysis approach.

### 4.4. Use of GLM for integrated meta-analysis

GLMs are commonly used in complex multi-factor designs to identify molecular changes in the context of development and/or adverse outcomes. For example, in transcriptomic profiling studies of primary cytotrophoblasts, we have applied linear models that account for dose, time, and/or genetic diversity to determine signatures associated with cell differentiation [18] or chemical toxicity [19]. These statistical approaches offer the ability to pinpoint specific changes due to the question of interest, while controlling for co-variates and potential confounders. In our current study, the second analytic approach involved integrating the datasets and using a two-factor GLM model to adjust for study-dependent effects across the three investigations. Our analysis revealed 174 genes to be DE in SPTB versus TD across the three studies. Trends in dysregulation were similar across all three studies, and, in general, genes tended to be downregulated. Of note, while patterns correlated across all three datasets, a higher concordance was observed between Chim et al. [12] and Leavey et al. [11], whereas Bukowski et al. [13] had less overlap with either of the other datasets. Our analysis highlights specific genes and pathways that may play roles in the initiation of SPTB and serve as biomarkers that can be applied in clinical investigations.

### 4.5. Challenges in identifying placental signatures of SPTB

The difficulty in identifying a common placental signature of SPTB is manifold. First, SPTB is a complex disease that encompasses a heterogeneous group of phenotypes with a diverse set of underlying mechanisms, including cervical softening, uterine overdistention, membrane weakening, activation of the fetal adrenal axis, and alterations in placental gene expression [20]. Second, whole placental tissue is comprised of a diverse group of cell types that have distinct functions and expression profiles. For example, global RNA profiling of separate subpopulations of placental trophoblast cells has demonstrated distinct cell-type-specific changes in expression with severe preterm preeclampsia [21]. Thus, using whole placental tissue may dampen the signals of individual cell types and produce results that are inaccurate and/or misleading. Third, there is heterogeneity in the techniques of placental sample handling, including timing of collection, size of tissue biopsy, and method of tissue preservation. Differences in these techniques can have a significant impact on the quality and stability of the gene expression profile [22]. Fourth, results may differ as a result of distinct methods of RNA profiling (e.g., microarray versus sequencing) and analytics, with prior reports suggesting that targeted expression profiling achieves higher coverage, evaluates a broader range, and identifies more true positives [23]. Finally, the identification of DE genes in SPTB is subjective of the comparison group. Genes implicated in SPTB have been identified in the context of a variety of “normal” controls (e.g.: term delivery without labor, medically-indicated (rather than spontaneous) PTB) or pregnancy complications (e.g., preeclampsia, fetal growth restriction)). As a first step, we chose to focus our analysis on placental expression of genes DE between SPTB versus labored TD due to the availability of datasets. Using alternative comparison groups would provide a different lens into the contributing factors of pregnancy complications.

### 4.6. Genes dysregulated in SPTB

Our subset of 174 dysregulated genes included genes implicated in placentation and SPTB. For example, within the top 20 most highly expressed genes (Fig. 2B), we identified genes with various roles in placental development, including (1) nidogen (*NID*), a key component of the extracellular matrix and human trophoblast cell adhesion [24]; (2) specificity protein 1 (*SP1*), a transcription factor that promotes trophoblast migration and invasion [25]; and (3) thrombospondin type I domain containing 7A (*THSD7A*), which modulates endothelial cell migration during placental angiogenesis [26].

In addition, we observed gene products implicated in SPTB, such as PPP1R414C, *RRM2B, SLIT2,* and *ADAMTS12. PPP1R14C* is a signal-transducing phosphatase that plays a role in regulation of myometrial contractility in preterm and term labor [27]. *RRM2B* is a DNA repair protein that has been shown to be upregulated in cases of SPTB compared to TD [28]. *SLIT2* is a glycoprotein that is linked to inflammation in SPTB [29]. *ADAMTS12* is a metalloproteinase and disintegrin that regulates trophoblast invasion and susceptibility to PTB [30]. We also identified new candidate biomarkers of SPTB including those linked with chromatin regulation and cellular stress (see below).

### 4.7. Chromosome regulation and SPTB

Chromatin modifications are associated with major steps in mammalian development, including placental growth and maturation [31]. Emerging studies suggest that placental development is largely regulated by changes in chromatin structure that enable greater access to transcriptional drivers [32-34]. For example, in human placentas, deacetylation of histone residues commonly associated with cytotrophoblast differentiation was found to be critical for development of the syncytiotrophoblast [35]. Acetylation patterns change over the course of pregnancy, with syncytiotrophoblast histone acetylation decreasing markedly from the first trimester to term [35]. Given the importance of chromatin regulation in placental development, perturbations in this pathway may underlie placentally-mediated pregnancy complications. For example, hyperacetylation of histone 3 lysine 27 acetylation (*H3K27ac*) in placental DNA regulatory regions is associated with upregulation of genes linked with fetal growth restriction [36], and hypermethylation of CpG site 14 of *VEGF* has been associated with PTB [[37].

In our analysis, we identified 24 placental genes associated with chromosome/chromatin organization that were significantly dysregulated in SPTB compared to TD. This subset of genes included molecules that play diverse roles in the regulation of chromatin structure and may function as biomarkers of SPTB. Our analysis validated previously identified biomarkers of SPTB, including *ZMYND8* and peptidylglycine *α*-amidating monooxygenase (*PAM*). A dual histone reader, *ZMYND8* has been found to be significantly downregulated in human SPTB versus TD placentas [38], similar to our results. PAM plays a role in condensation of the chromatin structure and has been found to be upregulated in association with SPTB [39]. Interestingly, our analysis demonstrated lower expression in SPTB vs. TD; this discrepancy may be related to the inclusion of both SPTB and preterm premature rupture of membranes and the use of alternative comparisons in the prior study [39]. Our study also revealed gene products that perform critical actions in chromatin organization but have yet to be described in the context of SPTB, including *KDM4C* and *CHMP2B. KDM4C* is a demethylase that regulates chromosome segregation and plays a key role in the expression of proliferation-associated genes in trophoblasts [40], and aberrations in trophoblast function are likely associated with SPTB. *CHMP2B* is a part of a large complex responsible for processing cell surface receptors and has been identified as part of the placental methylation profile associated with preeclampsia [41].

### 4.8. Cellular response to stress and SPTB

Cellular response to stress occurs in the setting of destructive alterations of both the intracellular and extracellular environment [42]. Aerobic environments contain high volumes of reactive oxygen species (ROS), introduced from either external sources or produced as a by-product of normal cellular metabolism [43]. Overwhelming ROS production that exceeds cellular antioxidant capabilities can cause severe oxidative stress, resulting in DNA damage, protein alterations, cell death, and ultimately placental dysfunction [44]. In pregnancy, oxygen metabolism with inherent production of ROS occurs in the placenta but is tightly regulated to maintain appropriate levels of ROS [45] Although ROS are required for healthy placental development and immunoregulation, ROS are also tied to the pathophysiology of various obstetric complications, including preeclampsia, preterm premature rupture of the membranes, and fetal growth restriction [45,46].

The DE genes identified here were enriched for biological processes connected with cellular response to stress, including a subset of 9 DE genes associated specifically with cellular response to decreased oxygen levels. This subset of genes encoded factors known to be involved in pathways that control cellular proliferation, cellular differentiation, and angiogenesis, including *SMAD4* and *EP300*. A signal transduction factor involved in tumor suppression and inhibition of epithelial cell proliferation, *SMAD4* is activated in human umbilical vein endothelial cells following exposure to hypoxia and interacts with hypoxia-inducible factor-1 (HIF-1) to modulate hypoxia-driven gene expression [47]. *EP300* also activates HIF-1 to stimulate hypoxia-induced genes and has been found to be increased in the serum of individuals with PTB compared to those with TD [48] Additional genes identified in this subset have yet to be explored in the context of SPTB and warrant further exploration. For example, VHL, a known regulator of HIF, is downregulated in the placentas of pregnancies complicated by preeclampsia [49] and may also play an important role in the pathophysiology of the placentally-mediated complication of SPTB.

### 4.9. Proposed regulators of transcriptomic signatures in SPTB

We assessed for potential upstream TF regulators of SPTB by performing enrichment analysis of TF-specific motifs in promoter regions of DE genes (Table 2). These investigations led to the identification of TFs that are suspected to play key roles in transcriptional regulation of the placenta. For example, NKX2-5 is a homeobox-containing TF that plays an important role in extra-embryonic development, and knockout of this TF in mouse models leads to abnormal vasculogenesis in the yolk sac [50]. In humans, placental NKX2-5 expression is significantly elevated in cases of severe preeclampsia [51], a complication of pregnancy that is mediated by the placenta. Another TF identified through this analysis is *ARID3A*, which is highly expressed in trophoblast cells and critical to placental development [52]. *ARID3A* knockout mouse models demonstrate abnormal placentation, fetal growth restriction, and upregulation of inflammatory mediators. Our meta-analysis was restricted to whole placental biopsies, but future studies can further explore these findings by interrogating single cell RNA placental profiles to identify particular placental cell types that may have regulatory roles in the development of SPTB. We predict that this approach will demonstrate that several of the proposed TFs are expressed in placental fibroblasts and endothelial cells and to a lesser extent in trophoblasts and Hofbauer cells.

### 4.10. Strengths and limitations

This study is limited by its reliance on publicly available data collected in prior studies. It is possible that these publicly available datasets do not represent the entirety of existing resources in the literature, due to publication bias and issues of open access. Furthermore, our results are inherently limited by any biases or errors contained in the original datasets. Clinical data are limited to those that were collected by the initial investigators, thus limiting our ability to provide a richer description of clinical characteristics of the included biospecimens. For example, while TD samples from studies A and B were specified as samples collected at delivery preceded by spontaneous labor, study C did not specify whether term samples were collected from those who experienced spontaneous labor or those who underwent induction of labor. This study is also limited in its examination of post-delivery placental samples, a limitation shared by similar studies. As a result, we cannot make inferences regarding the direction of association between the DE genes and SPTB, and it remains to be determined whether dysregulation of these genes is a cause or consequence of the disease state.

Despite these limitations, this study has numerous strengths. The major strength lies in the robust methods that incorporated both independent and aggregate analyses. This approach amplifies disease-specific signals, diminishes confounding signals, highlights the limitations of independent analyses, and serves as a template for future work in this area. Another strength of this study is the focus on the specific phenotype of SPTB preceded by spontaneous preterm labor, with a consistent control group of TD with labor. Because PTB is a heterogeneous disease, a better understanding of its etiology will require specific and separate analyses of the various phenotypes that lead to the common endpoint of premature delivery. Finally, this study highlights an important limitation of current work in the area – namely, that most publicly available transcriptomic datasets were too varied to include in aggregate analysis. In order to improve our understanding of the mechanisms underlying SPTB, future studies will require greater uniformity in study design and methodology.

## 5. Conclusion

Using aggregate analysis of publicly available transcriptomic datasets comparing SPTB to TD, this study identified 174 DE genes in the placenta across three unique studies. These genes were significantly enriched for chromatin organization and cellular response to stress and include previously proposed biomarkers of SPTB as well as new candidate biomarkers for SPTB. These results serve as promising preliminary findings that should be confirmed in larger populations in order to provide greater insight into SPTB, which remains a major complication of pregnancy with profound short- and long-term implications. Given the limitations of individual gene expression studies, efforts should be made to perform integrated analyses of previously published biomarker work to identify consistent signatures of SPTB. Further research is urgently needed to better understand the pathways contributing to this condition, with the goal of identifying a unique signature that can provide guidance for clinical efforts aimed at predicting, preventing, and ultimately treating SPTB.

## Supplementary Material

Supplemental Table 1

## Figures and Tables

**Table 1:** Summary of public datasets assessing placental transcriptome in the context of spontaneous preterm birth (SPTB) and term delivery (TD).

			SPTB (cases)					TD (controls)				
ID	GEO Dataset	Platform	n	Age (yrs)	GA (weeks)	BMI (kg/ m^2^)	CD	n	Age (yrs)	GA (weeks)	BMI (kg/ m^2^)	CD
A	GSE18809	Affymetrix Human Gene 1.0 ST Array	5	32	31.2	^a^	0	5	31	39.1	^a^	0
B	GSE73685	Affymetrix Human Gene 1.0 ST Array	2	^a^	^a^	^a^	2	5	^a^	^a^	^a^	5
C	GSE98224	Affymetrix Human Genome U133 Plus 2.0 Array	4	29.75	31.5	24.3	3	5	33	39.2	27.8	2

Abbreviations: BMI, body mass index; CD, cesarean delivery; GA: gestational age; SPTB, spontaneous preterm birth; TD, term delivery.

a Data unavailable.

**Table 2:** Transcription factor binding site (TFBS) enrichment analysis of differentially expressed placental genes in spontaneous preterm birth (SPTB) vs. term delivery (TD). Table displays transcription factors (TF) and associated JASPER identification (ID) annotations, TF family, total number of genes differentially expressed (DE) between SPTB vs. TD, and TFBS identified within DE gene subset. Fisher score signifies enrichment of the genes that contain the TFBS in the DE gene subset as compared to the total genome, and Z-score indicates enrichment of the TFBS in the DE subset as compared to the total genome. Based on the dataset (E-MTAB-6701)^a,b^, we included placental cells predicted to express TF in placenta (>0.5 transcripts per million) and highest transcript count in most highly expressed placental cell type (italics).

TF	ID	Family	DE genes	TFBS	Fisher score	Z score	Cells expressed (>0.5 TPM)	Highest (TPM)
NKX2-5	MA0063.1	Homeo	120 (22↑, 98↓)	991	28.7	20.9	*Mes*	5.2
PRRX2	MA0075.1	Homeo	106 (18↑, 88↓)	651	26.9	17.1	*Mes*, vasc, TB, Hof	29.0
SOX17	MA0078.1	HMG	99(22↑, 77↓)	349	25.7	11.5	*Vasc*; mes	137.4
HOXA5	MA0158.1	Homeo	122 (25↑, 97↓)	1038	24.5	19.9	*Vasc*: mes, Hof	2.0
SRY	MA0084.1	HMG	100 (20↑, 80↓)	533	23.7	19.6	*Vasc*, mes	3.3
TBP	MA0108.2	TATA-binding	75 (17↑, 58↓)	178	23.5	12.1	*mes*; vasc, TB, Hof	20.2
PDX1	MA0132.1	Homeo	105 (18↑, 87↓)	732	23.2	18.9	None	0.0
FOXA2	MA0047.2	Forkhead	79 (14↑, 65↓)	238	22.9	16.6	*Mes*	2.5
ARID3A	MA0151.1	Arid	108 (18↑, 90↓)	823	22.7	17.7	*TB*; mesc, vasc, Hof	378.8
NFATC2	MA0152.1	Rel	103 (22↑, 81↓)	495	21.6	10.1	Hof; vasc, mes, TB	51.3
GATA1	MA0035.2	GATA	96 (21↑, 75↓)	376	21.4	11.9	*Mes*; TB, Hof	23.1
FOXA1	MA0148.1	Forkhead	88 (15↑, 73↓)	330	21.2	15.3	*Vasc*	0.7
FOXQ1	MA0040.1	Forkhead	55 (9↑, 46↓)	114	21.0	10.7	None	0.0

Abbreviations: ↑, upregulated genes; ↓, downregulated genes; DE, differentially expressed; HMG, High Mobility Group; Hof, Hofbauer cells; mes, mesenchymal; TB, trophoblast; TF, transcription factor; TFBS, transcription factor binding site; TPM, transcripts per million; vasc, vascular/endothelial.

a Vento-Tormo R, Efremova M, Botting RA, Turco MY, Vento-Tormo M, Meyer KB et al. Single-cell reconstruction of the early maternal-fetal interface in humans. Nature. 2018; 563(7731):347–353. https://doi.org/10.1038/s41586-018-0698-6.

b Uhlen M, Oksvold P, Fagerberg L, Lundberg E, Jonasson K, Forsberg M et al. Towards a knowledge-based Human Protein Atlas. Nat Biotechnol. 2010; 28(12):1248–50. https://doi.org/10.1038/nbt1210-1248.
