# Supplementary material for: Integrated analysis of transcriptomic datasets to identify placental biomarkers of spontaneous preterm birth
Source: Placenta. Author manuscript; Available in PMC 2022 Dec 1. (PMC9715015; doi:10.1016/j.placenta.2022.03.122)
Supplement: Supplemental Table 1 [file NIHMS1843955-supplement-Supplemental_Table_1.docx]

**Supplemental Table 1**. Transcriptomic datasets related to spontaneous preterm birth (SPTB) and human placenta, identified on search of public repositories.

| Accession | Platform | Samples (n) | Tissue | Condition | Control |
| --- | --- | --- | --- | --- | --- |
| GSE18809 [1] | Affymetrix Human Genome U133 Plus 2.0 Array | 10 | Whole Placenta | SPTB | TD with labor |
| GSE98224 [12] | Affymetrix Human Gene 1.0 ST Array | 96 | Whole placenta | Term PE | TD |
| GSE73685 [3] | Affymetrix Human Gene 1.0 ST Array | 183 | Whole placenta  Decidua  Chorion  Amnion  Maternal blood  Myometrium | SPTB  PTB no labor | TD with labor  TD no labor |
| GSE73712 [4] | Illumina HiSeq 2500 | 30 | Villi trophoblast  Decidua | Infected PTB | TD no labor |
| GSE50879 [5] | Arraystar Human LncRNA microarray V2.0 | 40 | Cytotrophoblast | Preterm PE | PTB no infection |
| E-MTAB-5353 [6] | Illumina Human HT-12 WG-DASL | 36 | Decidua | PTB no labor | TD with labor  TD no labor |
| E-MTAB-5354 [7] | Illumina Human HT-12 WG-DASL | 24 | Cervix | PPROM | TD with labor  TD no labor |
| E-GEOD-9159 [8] | Affymetrix Human Genome U133 Plus 2.0 Array | 12 | Myometrium | PTB no labor | TD with labor  TD no labor |
| E-TABM-469 [9] | Exiqon miRCURY LNA microRNA Array v.8.1 | 30 | Chorioamniotic membranes | PTB with labor | TD with labor  TD no labor |
| GSE40182 [10] | Affymetrix Human Genome U133 Plus 2.0 Array | 39 | Cytotrophoblast | Preterm PE | PTB with labor |
| GSE14722 [11] | Affymetrix Human Genome U133A Array Affymetrix Human Genome U133B Array | 46 | Basal plate | Preterm PE | PTB no infection |
| GSE25861 [12] | Affymetrix Human Genome U133 Plus 2.0 Array | 10 | Vascular endothelial cells | PTFGR | PTB with labor |

PE, preeclampsia; PPROM, preterm premature rupture of membranes; PTFGR, preterm fetal growth restriction; TD, term delivery

**References**

1. Chim SSC, Lee WS, Ting YH, Chan OK, Lee SWY, Leung TY. Systematic identification of spontaneous preterm birth-associated RNA transcripts in maternal plasma. PLoS One. 2012;7(4):1–12.
2. Leavey K, Benton SJ, Grynspan D, Kingdom JC, Bainbridge SA, Cox BJ. Unsupervised Placental Gene Expression Profiling Identifies Clinically Relevant Subclasses of Human Preeclampsia. Hypertension. 2016;68(1):137–47.
3. Bukowski R, Sadovsky Y, Goodarzi H, Zhang H, Biggio JR, Varner M, et al. Onset of human preterm and term birth is related to unique inflammatory transcriptome profiles at the maternal fetal interface. PeerJ. 2017;2017(9).
4. Ackerman WE, Buhimschi IA, Eidem HR, Rinker DC, Rokas A, Rood K, et al. Comprehensive RNA profiling of villous trophoblast and decidua basalis in pregnancies complicated by preterm birth following intra-amniotic infection. Placenta. 2016;44:23–33.
5. Luo X, Pan J, Wang L, Wang P, Zhang M, Liu M, et al. Epigenetic regulation of lncRNA connects ubiquitin-proteasome system with infection-inflammation in preterm births and preterm premature rupture of membranes. BMC Pregnancy Childbirth. 2015;15(1):1–17
6. Rinaldi SF, Makieva S, Saunders PT, Rossi AG, Norman JE. Immune cell and transcriptomic analysis of the human decidua in term and preterm parturition. Mol Hum Reprod. 2017;23(10):708–24.
7. Makieva S, Dubicke A, Rinaldi SF, Fransson E, Ekman-Ordeberg G, Norman JE. The preterm cervix reveals a transcriptomic signature in the presence of premature prelabor rupture of membranes. Am J Obstet Gynecol. 2017;216(6):602.e1-602.e21.
8. Weiner CP, Mason CW, Dong Y, Buhimschi IA, Swaan PW, Buhimschi CS. Human effector/initiator gene sets that regulate myometrial contractility during term and preterm labor. Am J Obstet Gynecol [Internet]. 2010;202(5):474.e1-474.e20. Available from: http://dx.doi.org/10.1016/j.ajog.2010.02.034
9. D Montenegro, R Romero, SS Kim, AL Tarca, S Draghici, JP Kusanovic, JS Kim, DC Lee, O Erez, F Gotsch SH and CK. Immune activation and inflammation in HIV-1 infection : J Pathol. 2008;(October 2008):231–41.
10. Zhou Y, Gormley MJ, Hunkapiller NM, Kapidzic M, Stolyarov Y, Feng V, et al. Reversal of gene dysregulation in cultured cytotrophoblasts reveals possible causes of preeclampsia. J Clin Invest. 2013;123(7):2862–72.
11. Winn VD, Gormley M, Paquet AC, Kjaer-Sorensen K, Kramer A, Rumer KK, et al. Severe preeclampsia-related changes in gene expression at the maternal-fetal interface include sialic acid-binding immunoglobulin-like lectin-6 and pappalysin-2. Endocrinology. 2009;150(1):452–62.
12. Dunk CE, Roggensack AM, Cox B, Perkins JE, Senius F, Keating S, et al. A distinct microvascular endothelial gene expression profile in severe IUGR placentas. Placenta [Internet]. 2012;33(4):285–93. Available from: http://dx.doi.org/10.1016/j.placenta.2011.12.020
